# 2-Methyloxolane (2-MeOx) as Sustainable Lipophilic Solvent to Substitute Hexane for Green Extraction of Natural Products. Properties, Applications, and Perspectives

**DOI:** 10.3390/molecules25153417

**Published:** 2020-07-28

**Authors:** Vincent Rapinel, Ombéline Claux, Maryline Abert-Vian, Christine McAlinden, Mickael Bartier, Norbert Patouillard, Laurence Jacques, Farid Chemat

**Affiliations:** 1 Pennakem Europa, 224 avenue de la Dordogne, F-59944 Dunkerque, France; ombeline.claux@alumni.univ-avignon.fr (O.C.); Mickael.BARTIER@minakem.com (M.B.); npatouillard@pennakem.com (N.P.); laurence.jacques@minakem.com (L.J.); 2 GREEN Extraction Team, UMR408, INRA, Avignon University, F-84000 Avignon, France; maryline.vian@univ-avignon.fr; 3 Toxcel International Ltd., P.O. Box 93, Ledbury HR8 9JE, UK; Christine.McAlinden@toxcel.co.uk

**Keywords:** 2-methyloxolane, 2-MeOx, 2-MeTHF, bio-based solvent, green extraction, hexane

## Abstract

This review presents a complete picture of current knowledge on 2-methyloxolane (2-MeOx), a bio-based solvent for the extraction of natural products and food ingredients. It provides the necessary background about the properties of 2-MeOx, not only its solvent power and extraction efficiency, but its detailed toxicological profile and environmental impacts are discussed. We compared 2-MeOx with hexane which is the most used petroleum-based solvent for extraction of lipophilic natural products. The final part focuses on successful industrial transfer, including technologic, economic, and safety impacts. The replacement of petroleum-based solvents is a hot research topic, which affects several fields of modern plant-based chemistry. All the reported applications have shown that 2-MeOx is an environmentally and economically viable alternative to conventional petroleum-based solvents for extraction of lipophilic foodstuff and natural products.

## 1. Introduction

During the 20th century, extraction solvents have been exclusively volatile organic compounds (VOCs) obtained from non-renewable resources, and now, most of them are known or suspected to be harmful to both human health and the environment. One widely used solvent (1500 ktons/year) is hexane, a mixture of C6 products obtained from the distillation of petroleum mixtures. The primary advantage of such a petroleum based solvent is the ease of production (low cost) and chemical properties that impart ideal functionalities, particularly its ability to solubilise a variety of products, including vegetable oils, aromas, or colors. Nevertheless, hexane is a neurotoxic substance, classified as a reproductive toxicant (category 2) and is toxic for the aquatic environment (category 2), under the European Directives and Registration, Evaluation, Authorization and Restriction of Chemicals (REACH) regulation.

Extraction of natural products is often considered as “clean” or “green” when compared with traditional chemical industries, but researchers and professional specialists know that environmental, security, and health impacts can be greater than first appeared. For example, 1 kg of rose absolute (refined perfume extract) does not only require almost 2 tons of fresh roses as raw material, but also a large quantity of petroleum based solvent (hexane), a lot of energy (mainly fossil) for extraction and evaporation, and water to cool and clean, on top of generating toxic solid waste with petroleum-based solvent residues, wastewater, and emissions of VOCs. As another example, to extract 1 ton of rapeseed or soybean oil, we need more than 4 tons of raw material to be processed with almost same amount of hexane. Despite an optimized solvent recycling, it is known that up to 1 kg of solvent can be lost per ton of raw material. Addition of fresh solvent is necessary to compensate losses due to leakages to the atmosphere (2/3) or solvent traces in final products (1/3), such as oils or feed meals. If the hexane residual is limited to 1 ppm in refined oil within the European Union, no regulatory limit currently exists for feed meal.

The choice of the extraction solvent is crucial, and a number of criteria must be considered (properties, toxicity, cost, etc.). The recent need for greener products led to the publication of the twelve principles of green chemistry [1] and the twelve principles of green engineering [2], which define the good practices to be adopted. Following those principles, researchers from academia and industry defined the term of “green extraction” and established the six principles of green extraction [3]. The choice of the solvent for green extraction must particularly ensure the durability of global process and good practice guidelines must be respected, such as:

1. Use of 100% natural, natural origin, renewable or agro-sourced solvent with condition of having good knowledge, evaluation, and control of related risks
2. Avoid the use of solvent which might affect the safety and health of operators and consumers: no CMR, toxic, allergens, endocrine disruptors substances
3. Use of solvent suitable with existing industrial facilities
4. Prefer a solvent with high rate of recyclability, high bio-degradability and no bio-accumulation, to limit global process impact on environment
5. Use of solvent with low VOC emissions related
6. Use of solvent which limits energy consumption and cost of global process
7. Ensure a maximal solvent recovery using various available techniques

In this context, this review presents a complete picture of current knowledge on 2-metyloxolane (2-MeOx)—also known as 2-methyltetrahydrofuran (2-MeTHF)—as a bio-based solvent for the extraction of natural products. Readers such as chemists, biochemists, chemical engineers, physicians, and food technologists, from academia or industry, will find a deep and complete perspective regarding 2-MeOx properties, applications, toxicological, and environmental impacts, and also industrial applications. The first part presents history, synthesis, sourcing, and production of 2-MeOx. The second part is dedicated to the physico-chemical properties and solvation power comparing 2-MeOx with hexane. The third part focuses on applications of 2-MeOx as solvent of different targeted natural products and presentation of the most relevant extraction procedures. The last parts give new insights in term of toxicity and regulations, even for operators or final consumers, up-scaling and industrial applications, quality, security and safety considerations, economic and environmental impacts, and future directions for research and industry.

## 2. Production of 2-Methyloxolane

2-Methyloxolane (2-MeOx) is a cyclic ether, issued from carbohydrates derived from lignocellulosic biomass, which represents the most abundant biomass resources on earth [4]. The term lignocellulosic covers a range of biomass containing cellulose, hemicellulose (polysaccharides) and lignin (aromatic polymer), making a rigid, compact, and complex assembly of polymers naturally recalcitrant to microbial and enzymatic degradation [5]. The content of cellulose is generally in the range of 29–45%, and hemicellulose in the range of 18–30%. Industrially, 2-MeOx is produced from agricultural by-products such as corn stover, sugarcane bagasse, and rice straw, which could be found mainly in China, South Africa, and Dominican Republic. Agricultural by-products as lignocellulosic biomass are preferred, because they do not compete with the use of land for the production of food [6].

**Figure 1:**
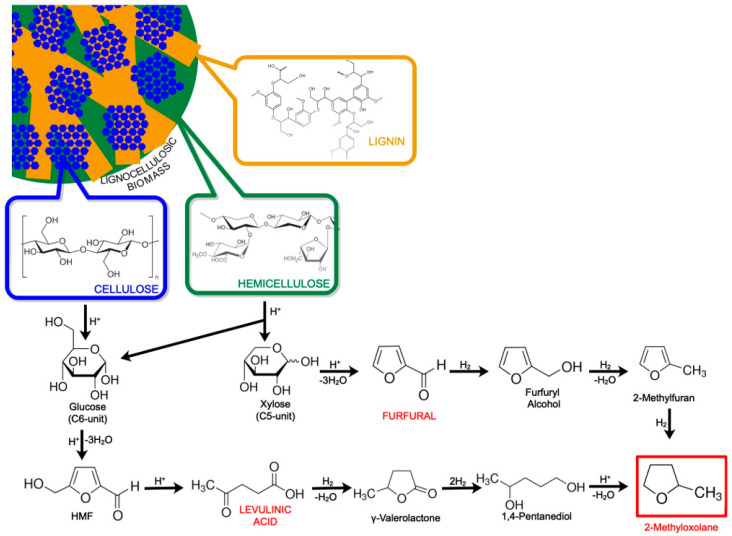
Production of 2-methyloxolane (2-MeOx).

First, harsh acidic pretreatment is required for deconstructing lignocellulose to make polysaccharides into more accessible intermediate sugars for subsequent conversions [7]. After separation of the solid residue (from lignin), the acidic solution containing a mixture of both hemicellulose and cellulose is subject to hydrolysis of the polymers into monomeric C5 (pentoses) and C6 (hexoses) sugars. Then, C6- and C5- monosaccharides undergo multiple acid-catalyzed reactions to give the platform molecules levulinic acid (LA) and furfural (FAL), which are used as the building blocks for the synthesis of 2-MeOx. Indeed, 2-MeOx can be produced either from FAL pathway or LA pathway, as shown in Figure 1. Hayes et al. reported that the “biofine” process [8] seems to be the only industrially relevant process able to lead the production of either LA or FAL starting from a mixture of C5 and C6 monosaccharides [6].

### 2.1. Furfural Pathway

FAL is a colorless, organic liquid aldehyde (C_5_H_4_O_2_) obtained by dehydrating C5 sugars. It naturally occurs in small quantities in many foods such as fruits, cocoa, in tea, coffee (55–255 mg/kg) and whole grain bread (26 mg/kg) [9]. It has been primarily used as a solvent for extraction of heavy molecules in oil refineries [10], but it rapidly became a renewable building block for industrial downstream products such as resins and polymers, solvents, lubricants, and fuel additives. Finally, due to its desirable aromatic characteristics, caramel smell, it is used to a lesser degree as a flavoring agent in a variety of food products and alcoholic and non-alcoholic beverages [11].

In the synthesis of 2-MeOx, two successive hydrogenations of FAL over Cu-Zn catalysts allow a nearly complete conversion to 2-methylfuran in a range of temperature of 200–300 °C, with a yield >95% [12]; 2-methylfuran is then isolated by distillation and converted into 2-MeOx at lower temperature (100 °C) over Ni catalyst with a yield around 85%. The 2-MeOx is also recovered by distillation, which allows the production of a product purity >99.9%. The choice of the catalyst has a great importance in the conversion yield as a wide variety of compounds can be obtained depending on the reaction conditions [12].

### 2.2. Levulinic Acid Pathway

LA is a keto acid (C_5_H_8_O_3_) found as white crystalline solid. It is a highly versatile compound used for several applications like in resins or plasticizers industry, but also as precursor for pharmaceuticals and others high-value added chemicals [13,14,15]. LA is considered as one of the United States Department of Energy’s (DOE’s) top 12 platform bio-based chemicals [16].

The synthesis of 2-MeOx from LA consists in consecutive catalyzed hydrogenations and dehydrations. The catalyzed hydrogenation of the keto group of LA leads to a hydroxyl acid that results in γ-valerolactone. Further hydrogenation of the keto bond of γ-valerolactone allows the formation of the cyclic hemiacetal in equilibrium with the aliphatic hydroxyl aldehyde. The hydrogenation of the last carbonyl group leads to 1,4-pentanediol that can be etherified in 2-MeOx by dehydration in acid conditions. Although the 2-MeOx can be directly produced from LA using a batch-type reactor, harsh reaction conditions and/or precious metal catalysts such as Ru [17], Pt [18] and Pd [19] are required. Using these catalysts, the conversion of LA to 2-MeOx gives 85–92% yields.

### 2.3. History

**Figure 2:**
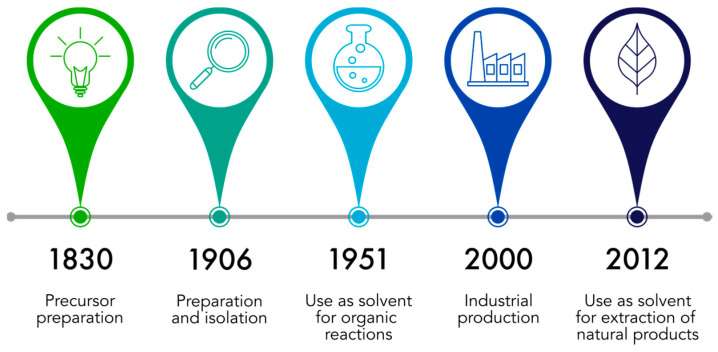
Chronological milestone of 2-MeOx production.

To the best of our knowledge, 2-MeOx was first prepared and isolated in 1906 [20], during research on furfural, which was discovered 76 years before (Figure 2). At that time, there were plenty of resources available to produce furfural and researchers were trying to find uses for it, either directly or through an impressive number of derivatives [21]. Due to its interesting properties, 2-MeOx was first employed as a reaction medium in 1951 in laboratories [22]. Almost 100 years were necessary between its first isolation and its efficient production at an industrial scale [23]. It is only a few years ago, in 2012, that 2-MeOx began to draw the attention of researchers working on the extraction of natural products while searching for alternative to benchmark petrochemical solvents.

## 3. Solvent Properties

The general properties of 2-MeOx and hexane (food extraction grade) are summarized in Table 1. 2-methyloxolane (also known as 2-methyltetrahydrofuran or 2-MeTHF), is a clear liquid with an ether smell, produced from crop by-products. It is currently mainly used as solvent for chemical reactions as a green alternative to tetrahydrofuran (THF) [24,25].

The properties that make a solvent a “good extraction solvent” are complex and must fulfil many criteria (technical, economic, environmental, regulatory, etc.). From a purely technical point of view, hexane is a good reference for extraction of lipophilic products, because of its advantageous properties: boiling point around 70 °C, good chemical selectivity towards lipids, high volatility, easy separation with water, etc. However, it must be noted that extraction industrial processes have been optimized since the 1950s with this solvent, therefore only solvents with close properties could be easily transposed at an industrial scale, in particular for the oil and fats industry that require large plants. In this context, 2-MeOx shows interesting properties: briefly, its boiling point (80 °C) is high enough to allow a good extraction temperature, but low enough to be easily removed from final products and recycled. In addition, the density (0.855) and viscosity (0.6 cP) are close to hexane and in the acceptable ranges for efficient diffusion through solids particles.

In order to better understand the properties that make a solvent a good extraction solvent, we can decompose the solid-liquid extraction process in several steps involving the solvent:

- Solvent diffusion into the solid
- Solubilization of the solute in the solvent
- Solvent + solute diffusion out from the solid
- Solute isolation by solvent evaporation

### 3.1. Diffusion

The first step generally considered in the solid-liquid extraction is the solvent diffusion from the external medium into the matrix. The diffusion steps (in and out) are usually the limiting steps of the extraction process and are essentially influenced by the temperature and the structure of the solid particle (porosity, moisture, bulk density, type of cell membranes, etc.). Therefore, the solvent itself as usually only a limited influence on the speed of diffusion, but we can say that mass diffusion is faster for low viscosity and water miscible solvent (if moist material). In the case of 2-MeOx, viscosity is a little bit higher, but still close to hexane viscosity (0.6 cP vs. 0.3 cP). However, in contrast with hexane, the partial water miscibility of 2-MeOx may result in a better diffusion in cases when the solids to extract contains moisture.

### 3.2. Solubilization

Once the solvent is inside the solid, the solvation phenomenon occurs. Due to its particular structure, 2-MeOx is mostly lipophilic (log *P* = 1.85) therefore it is able, to solubilize both fatty molecules, like hexane (log *P* = 4.00), but also more polar molecules thanks to the oxygen atom (dipole moment = 1.38 D). Beyond those classical indicators, thermodynamics are now used to predict more precisely the solubilization capacity of a solvent. In the context of vegetable extraction, two models are mostly used since 2010: Hansen Solubility Parameters (HSP) and COSMO-RS models [28,29,30,31,32].

Hansen solubility parameters are already quite known by researchers to describe and quantify the various types of interactions between a solvent and a solute (dispersion δd, hydrogen bond δh, and polar bond δp) [33]. In the case of 2-MeOX, Hansen solubility parameters show that, unlike hexane (δh = 0; δp = 0), interactions with more polar compounds via hydrogen and polar bonding are possible. 2-MeOx parameters (δh = 4.8; δp = 4.6) are close to chloroform (δh = 3.1; δp = 5.7), known to be used for solubilization of fatty compounds in analytical methods [34]. Interestingly, the polar bonding contribution is higher for 2-MeOx, highlighting the potential for solubilization of more polar molecules.

**Figure 3:**
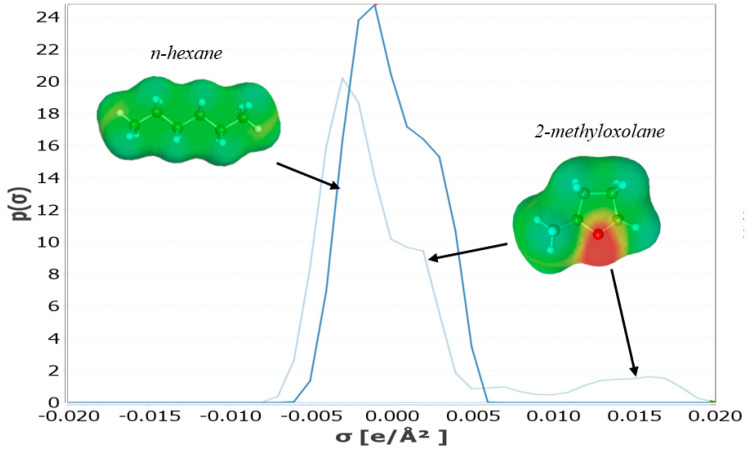
σ-surfaces and σ-profiles of 2-MeOx vs. *n*-hexane. Yellow/Red: molecule charge density <0, Green: molecule charge density = 0 [36].

HSP predictions are more and more superseded by COSMO-RS simulations, using both quantic and thermodynamic calculations for predictions of physico-chemical properties, including solubilization [30,32,35]. Figure 3 illustrates for *n*-hexane and 2-MeOx the polarization charge density around the molecule (σ-surface) and its distribution (σ-profile). The polarization charge density σ is a good local descriptor of the molecular surface polarity. Green surfaces that are visible in the σ-surfaces of both *n*-hexane and 2-MeOx are neutral surfaces (fatty chains) that interacts mostly with other neutral surfaces such as fatty chains in fatty acids. On the contrary, the red surface on 2-MeOx reveals the presence of negative polar surface in the molecule that may interact with positive polar surfaces of solutes. Both σ-surface and σ-profile are the starting points used by the COSMO-RS model for solubility predictions. In particular, COSMO-RS is able to predict absolute or relative solubilities of a solute in several solvents or of several solutes in a solvent. In most cases, absolutes solubilities, expressed as “x_solub_” values going from 0% (fully insoluble) to 100% (fully miscible), are used to compare the solubility of a specific solute in several solvent. Examples of solubility calculations for some lipophilic solutes (oil components, carotenoids, terpenes, etc.) in *n*-hexane and 2-MeOx will be later presented in part 4.

### 3.3. Solvent Distillation

Once the target compounds are solubilized in the solvent and extracted from the solid, the mixture is usually separated by filtration or centrifugation to get two fractions: the extracted solid and the extract solution which will be distillated to get the dry extract. This distillation step is generally the most energy consuming step, as a large quantity of solvent must be heated until the boiling point and then vaporized (see Equation (1)). Therefore, an ideal extraction solvent must have a low boiling point, low Cp (heat capacity) and ∆H_vap_ (vaporization enthalpy).

(1) $$∆E_{mass}=E_{heat}+E_{vap}=C_{p,m}\times \left(T_{b}-T_{0}\right)+∆H_{vap}$$

With: ∆*E_mass_* the total energy consumption for distillation by kg for solvent; *E_hea_*_t_ the energy consumption for heating 1 kg of solvent of heat capacity *C_p,m_*_,_ from the starting temperature T_0_ to the boiling point *T_b_*; *E_vap_* the energy consumption by kg for solvent vaporization; ∆*H_vap_* the vaporization enthalpy of the solvent.

2-MeOx (excluding azeotropes) has a higher boiling point and vaporization enthalpy than hexane; therefore higher energy consumption is expected for solvent distillation step. However, it is still acceptable for industrial applications and still much lower than it would be with ethanol (∆*H_vap_* = 843 kJ/kg), which is the other viable solvent considered for oil extraction.

### 3.4. Safety

As with many organic solvents, 2-MeOx is a flammable liquid therefore it must be handled with caution at laboratory or industrial scales. Even if its flash point is higher than hexane (−11 vs −30 °C), it is still far lower than storage and process temperatures, so the risk is the same. Moreover, the explosivity interval is a bit larger than for hexane (1.5–8.9% vs 1.1–7.4%), however the lower explosivity limits (LEL) is higher, meaning that more solvent vapors are necessary for ignition than for hexane. It must be also noted that 2-MeOx electrical conductivity is much higher than hexane, thus reducing the risk of electrical charge accumulation in the piping during solvent flowing [37]. However, in any case, both solvents must be handled in explosion-proof conditions, in agreement with local regulations. Interestingly, it must be also noted that the strong ether odor, usually seen as a major drawback of 2-MeOx, is actually a “embedded safety” as workers can immediately detect the presence of solvent vapors (odor threshold <1mg/m^3^) way before it reaches the LEL or hazardous exposure levels. Moreover, any vapors of solvent in working atmosphere can be very easily scrubbed using water sprinkling thanks to 2-MeOx solubility in water (14% at 20 °C).

In parallel, like most of the ethers, 2-MeOx is prompt to generate peroxides in presence of oxidative species, with a formation kinetic close to THF [24]. For this reason, 2-MeOx should be always stored away from light, with peroxide inhibitors (preferentially natural α-tocopherol) and/or under a nitrogen blanket. In the context of natural product extraction processes, we do not expect major issues related to peroxides as (i) extractions will be run away from the light and from air (for flammability reasons), usually at moderate temperature (<80 °C) during a limited time (generally less than 4 h), (ii) vegetable material usually contains natural antioxidant such as phenolic compounds, carotenoids, tocopherols etc. that would inhibit peroxides formation, and (iii) solvent distillation is never supposed to reach dryness (presence of the vegetable extract), reducing the risk of peroxides concentration, if any.

## 4. 2-Methyloxolane as Extraction Solvent for Production of Natural Extracts

### 4.1. Flavours and Fragrances

Flavors and fragrances are complex mixtures of a huge number (sometimes hundreds) of volatile compounds responsible for the organoleptic properties of a product. It is important to note that flavors do not have any nutritional value, but they are still essential in our alimentation, for example to stimulate appetite or can enable the distinction between fresh and rotten food. These aromatic compounds are small molecules made of a linear, cyclic, or aromatic hydrocarbon skeleton. Among them, terpenes are a preponderant category of lipophilic aromatic molecules. They can be simple hydrocarbons (limonene, myrcene), alcohols (linalool, menthol), aldehydes (geranial), or ketones (carvone). Even though chemistry grants access to a wide range of synthetic aromas, the appearing current trend of consumers is to ask for natural products which are believed to be healthier and eco-friendly [38]. Natural flavors and fragrances can be found in an impressive variety of plants, originating from different parts: flowers, leaves, berries, fruit peels, buds, seeds, bark, roots, etc.

Industrially, two extraction processes are commonly used to recover aromatic compounds from plants: Hydrodistillation and solvent extraction. The first method consists of exposing the matrix to steam in order to extract the volatile compounds enclosed in the plant cells. That fraction is called an essential oil. However, this technique involves working at high temperature and is not compatible with heat-sensitive molecules. With solvent extraction, it is possible to work at lower temperatures. However, the crude extract (named concrete) often contains other compounds than the volatile ones and needs to be purified with alcohol to recover only the odorous fraction, giving an “absolute”. When extracting with a solvent, its nature is really important, as it will directly impact the composition of the extract and its quality. Indeed, sensitive molecules can be deteriorated when evaporating the solvent; solvent which can also be difficult to eliminate completely afterwards. Hexane is typically one of the main solvents used to recover aromatic compounds from natural products. Considering its hazardous characteristic to human health and environment, a lot of efforts are made to find a suitable alternative applicable in the industry [30,31,39,40].

**Figure 4:**
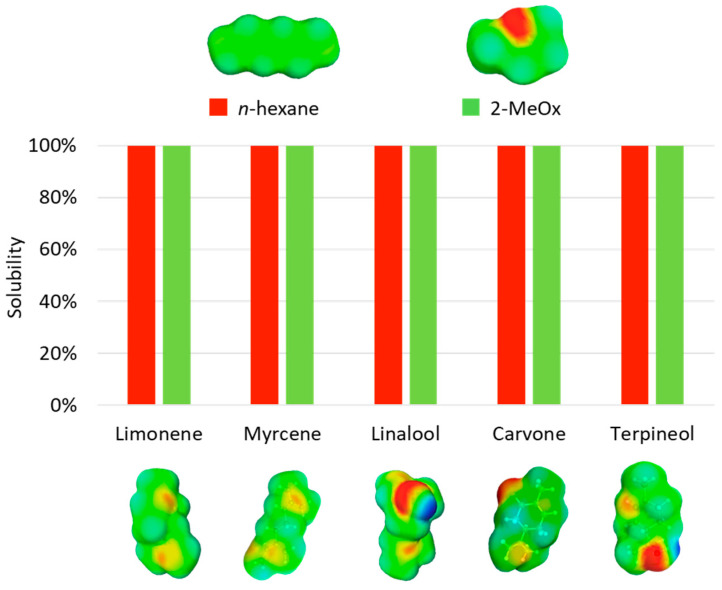
Representation of the predicted solubilities of some aroma compounds in *n*-hexane and 2-MeOx and their corresponding sigma surfaces, given by COSMO-RS. Rating: 0–20% bad solvent; 20–60% average solvent; 60–100% good solvent.

To help scientists to find a convenient solvent, predictive tools such as the Hansen Solubility Parameters or COSMO-RS have been used to evaluate the solvation of a molecule in a solvent. To illustrate, the solubilities of several aroma compounds in *n*-hexane (main isomer of extraction-grade hexane) and 2-MeOx were calculated using COMO-RS and are shown in Figure 4. These results indicate that both *n*-hexane and 2-MeOx can effectively solubilize aromatic molecules such as limonene or carvone. However, the efficiency of extraction of compounds from a matrix is a complex process that cannot be simulated considering only the solvation parameter. Therefore, the results given by predictive tools are only the first step to find the best solvent and have to be combined with experimental studies. Recent works combining both approaches for hexane substitution by 2-MeOx are reported in Table 2.

In 2014, Filly et al. compared the performances of nine solvents to *n-*hexane in order to extract aromas from blackcurrant buds [29]. This study was based on theory (Hansen Solubility Parameters) and experiments. Eventually, it was concluded that 2-MeOx was the most suitable solvent to replace hexane for the extraction of these aromas, giving an extract with similar chemical composition. Some of the main components found in both concretes were δ-3-carene, terpinolene, sabinene, and β-caryophyllene, suggesting that 2-MeOx enables the extraction of non-oxygenated terpenes as well as hexane does. It is interesting to note that the crude yield reached with 2-MeOx was twice as high as with *n-*hexane (7.10 and 3.87% respectively); the authors concluded that this is due to the extraction of other molecules than aromas from the matrix. The next year, they assessed the extraction of carvone and limonene from caraway seeds [30]. This investigation relied again on the use of a predictive tool combined with experiments. This time, the criteria applied to select the best solvents were the selectivity of carvone against limonene and the concordance with the chemical composition of the essential oil, recovered through Clevenger hydrodistillation. Therefore, 2-MeOx was not identified as an outstanding solvent, even if it afforded the best limonene recovery and an extract with a chemical composition close to the *n-*hexane one. In 2019, 2-MeOx was again tested, after a COSMO-RS evaluation, to recover flavors from lentil leaves by Chaabani et al. [41]. Some major compounds of the 2-MeOx extract (β-caryophyllene, germacrene D, β-elemene, and α-pinene) were also abundant in the hexane one. On this matrix, 2-MeOx gave the third highest yield among the green solvent tested. The same year, a study conducted by Ozturk et al. showed that 2-MeOx was a promising solvent for the extraction of limonene from orange peel residues [42]. At optimum conditions, its outperformed hexane by increasing limonene extraction yield by 40%. In addition, the solvent was successfully recycled within the process, and it provided 2-fold limonene extraction yields in comparison to that provided by hexane after three consecutive extraction cycles. The results of this analysis support the suitability of replacing hexane with 2-MeOx to develop sustainable processes. Recently, we investigated the potential of using 2-MeOx for the extraction of volatile compounds from hop cones [40]. Again, the crude yields provided by 2-MeOx either after maceration or Soxhlet procedure were higher than that of hexane (16.6 and 20.2% against 12.7 and 17.9%). Besides, both extracts showed relatively similar chemical and olfactory profiles.

These theoretical and experimental works proved that 2-MeOx can recover of the same aroma compounds than hexane (mainly terpenes, either oxygenated or not). However, their proportions can differ, and the extracts given by this new solvent can contain other compounds. A classical procedure to extract aroma compounds with 2-MeOx is described by Filly et al. [30]. First, the dried plant material is ground to a suitable size. Then, 5 g of raw material are placed into a flask filled with 50 mL of solvent. The extraction is performed using a conventionally heated reflux equipment at the boiling point of 2-MeOx for two hours. Then, the mixture is filtered, and the solvent eliminated through evaporation under vacuum. It is worth to note that the chemical composition of an extract produced with this new solvent is likely to be different than hexane extracts. Thus, it would be relevant to identify a convenient purification method and realize sensory analyses to figure out if the new extract is similar to the conventional one or if it should be considered a brand-new product.

### 4.2. Colors

The history of colors goes back to the time when men started to employ them in order to color themselves or represent the world, they were living in. Until the end of the 19th century, plant colors have been one of the most important sources of coloring material. After some works on plant chemistry, the first total chemical syntheses of colors were performed successfully. These works and progress in organic chemistry marked the advent of industrial color synthesis and the decline of vegetable coloring matters. Nowadays, color additives are still widely used to enhance the aesthetic value of foods, but they are mainly artificial and made from petrochemical sources. However, even if regulated, these synthetic substances are suspected to release harmful chemicals for the environment [43]. On the contrary, natural colors constitute renewable resources and can meet the consumer expectation for sustainable food. The colors that we can see in nature are principally due to the presence of one or several of the following compounds: flavonoids and especially anthocyanins, responsible for the red, purple, or blue in berries; chlorophyll, giving the green color of almost all plant leaves; quinones, for example alizarin which has a deep red color, indigo dyes, carotenoids, imparting a yellow, orange or red color in carrots, oranges, tomatoes, red peppers, etc.

In particular, carotenoids are currently one of the main groups of natural colors that are widely used in the industry. They can act as food additives, cosmetic colorants, antioxidants, and are also precursors of vitamin A [44]. Carotenoids can be divided as carotenes, which are exclusively hydrocarbons, and xanthophylls. This wide category of compounds can be synthesized by many plants, algae and bacteria. Due to their hydrophobicity, their extraction is usually achieved with petrochemical organic solvents and especially hexane [45]. Considering its hazardousness and because of an increase in demand for natural products, alternative extraction methods are currently under research.

**Figure 5:**
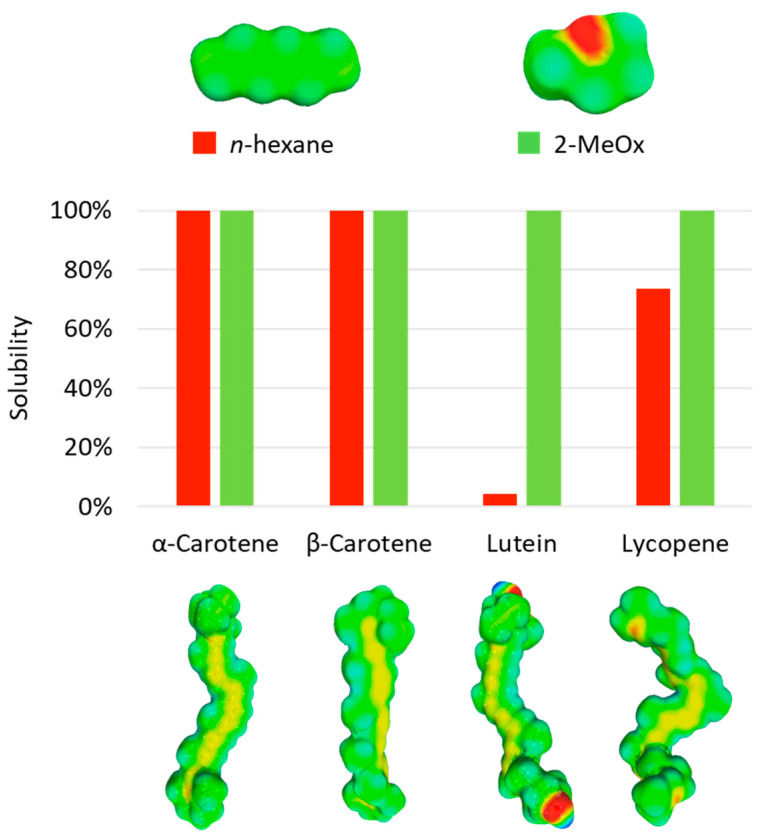
Representation of predicted solubilities of some carotenoids in *n*-hexane and 2-MeOx from [46] and their corresponding sigma surfaces, given by COSMO-RS. Rating: 0–20% bad solvent; 20–60% average solvent; 60–100% good solvent.

Thanks to predictive tools, the solvation of color compounds in a solvent can be assessed in order to find a suitable candidate to replace hexane for their extraction. The solvation probabilities of four usual carotenoids in *n*-hexane and 2-MeOx were calculated by Yara-Varón et al. using COMO-RS (Figure 5) [46]. These results indicate that 2-MeOx could be as good as *n*-hexane in solubilizing carotenoids, and even better than the reference for slightly polar molecules like lutein. However, again, the predicted solvation performances of solvents are not sufficient to make a choice and they have to be confirmed with experiments. Given the encouraging results obtained with predictive tools, the possible use of 2-MeOx to replace hexane for the extraction of carotenoids has been recently evaluated, as reported in Table 3.

Sicaire et al. conducted a kinetic study of the extraction of carotenoids from dried carrots with *n-*hexane and 2-MeOx in 2014 [25]. As a result, they found that both starting accessibility (quantity of solute directly available from the surface of the matrix) and effective diffusivity (coefficient representing the speed of diffusivity of the solute in the solvent) of color compounds were higher in 2-MeOx, resulting in a faster extraction. Quantitatively speaking, 2-MeOx extracted 23% more carotenoids than *n-*hexane did after six hours of extraction. The same trend was obtained by Yara-Varón et al., who evaluated 2-MeOx among five green solvents for the substitution of *n-*hexane in the extraction of carotenoids from the same plant matrix [46]. In accordance with COSMO-RS predictions, one of the highest yields was obtained using 2-MeOx, with 12% more carotenoids extracted compared with *n-*hexane after one hour.

The potential of 2-MeOx was also assessed to extract carotenoids from algae. In 2017, Damergi et al. obtained good results in extracting wet *Chlorella vulgaris* with pure 2-MeOx for 30 min, at 110 °C and under pressure (45% of total carotenoids extracted) [47]. However, the optimum conditions were found to be with dry biomass and a 1:1 mixture of 2-MeOx and ethanol. Here, 2-MeOx enabled the recovery of both carotenes and xantophylls, like astaxanthin or lutein. Besides, the different crude yields were way higher than the actual carotenoid yields, meaning that other compounds (probably oil and waxes) were extracted from the algae together with carotenoids. In 2019, Samorì et al. extracted *Haematococcus pluvialis* cultures with several green solvents [48]. Among them, 2-MeOx was one of the most effective, giving excellent astaxanthin recovery (>80%) in only 30 min. A patent has even been granted to Reddy et al. from Western Washington University describing the extraction of fucoxanthin and other carotenoids from defatted marine algae using 2-MeOx [49].

These different studies showed that 2-MeOx can be at least as effective as hexane to extract carotenoids from plants or microalgae. It allows the recovery of both apolar carotenes and more polar xantophylls. In addition, a kinetic study showed that the extraction process was faster with 2-MeOx than with hexane. Nevertheless, it appears that 2-MeOx is not selective towards carotenoids and can extracts other compounds, depending on the initial composition of the matrix, in this case using co-solvents such as alcohols can be relevant. A classical procedure to extract carotenoids with 2-MeOx is described by Yara-Varón et al. [46]: First, the dried plant material is ground to a suitable size immediately before extraction. Then, 30 g of raw material are placed into a jacketed reactor filled with 120 mL of solvent. The mixture is mechanically stirred and extraction is performed in the dark, at 65 °C for one hour. Then, the mixture is filtered and the solvent eliminated through evaporation under vacuum.

### 4.3. Lipids

Lipid is the general term for biomolecules that dissolve in non-polar solvent, comprising oils and fats. Although “fats” is often used as a synonym of lipids, it normally refers to lipids which are solid at room temperature, while oils are fluid. Lipids are essential for human life as, together with carbohydrates and proteins, they are one of the three macronutrients used to produce energy by the body through oxidation process [50]. This energy is crucial, as it ensures normal functions of vital organs (brain, heart, or lungs) and enables any physical activity. They also have other biological roles, as they are important constituents of cell membranes, structural components of hormones, and play a part in cell signalization. Four lipid subgroups are particularly important from a biological point of view: fatty acids or free fatty acids (FFAs), triacyl glycerides (TAGs), phospholipids, and sterols.

A fatty acid is a carboxylic acid with a long aliphatic chain, either saturated or unsaturated. Some fatty acids, such as α-linolenic and linoleic acids, are called essential, because they are crucial for humans and animals health, as they are not able to synthesize them. In fats or oils, fatty acids can be found as glycerol esters formed of one, two, or three fatty acid chains: monoacylglycerides (MAGs), diacylglycerides (DAGs), and triacylglycerides (TAGs). TAGs are generally the main component of vegetable oils, while other organisms like algae or insects can contain FFAs, MAGs, DAGs, and TAGs in varying proportions. Phospholipids are made of two fatty acids bound to the hydrophilic glycerol-3-phosphate. Because of their amphiphilic characteristic, phospholipids like phosphatidylcholine (PC) are an important constituent of cell membranes [51]. Sterols are a subgroup of steroids which are notably involved in the formation of various hormones, such as testosterone and estrogen. In addition, fatty substances can also contain fat soluble vitamins such as tocopherols (Vitamin E).

Regarding their essential role in animal diet, most of the lipids produced today are used for food and feed. However, their properties make them interesting for several industrial applications: as lubricants, binding agent in paintings, active ingredient, or texturizing agents in cosmetics, etc. Moreover, the idea of using oils to produce biodiesel recently emerged, with the aim of finding a convenient alternative to fossil fuels. Depending on the biomass they come from, biofuels can be classified into different generations. Each one presents advantages but also has drawbacks [52]. First generation biodiesels were made from edible crops oil, while the second and third generation biodiesel are based on the use of non-edible plant oil and microalgae, respectively. According to recent estimations, the world production of vegetable oil could reach 214 Mt in 2020, of which 143 Mt (67%) would be used for food and 30 Mt (14%) for biofuel production [53]. Industrially, two different processes can be employed to extract oil, depending on seed’s oil content. If it is higher than 25%, seeds are usually mechanically pressed to collect 2/3 of the total oil content. However, when the oil content in the seed (or in the press cakes) is less than 25% the product often undergoes solvent extraction. Again, hexane is still conventionally the solvent used in the oil industry despite its drawbacks. Considering its hazardous characteristic to human health and environment, researchers started an intensive search for suitable alternative solvents since the 1980s [54]. They tested several alternatives such as ethanol, isopropanol, and supercritical CO_2_ or even liquefied gases; however, none of them were as efficient and economic than hexane, a reason why it is still used today [55]. Only later in the 2010s, few studies came out about oil extraction using “green” solvents, including 2-MeOx [25,31].

**Figure 6:**
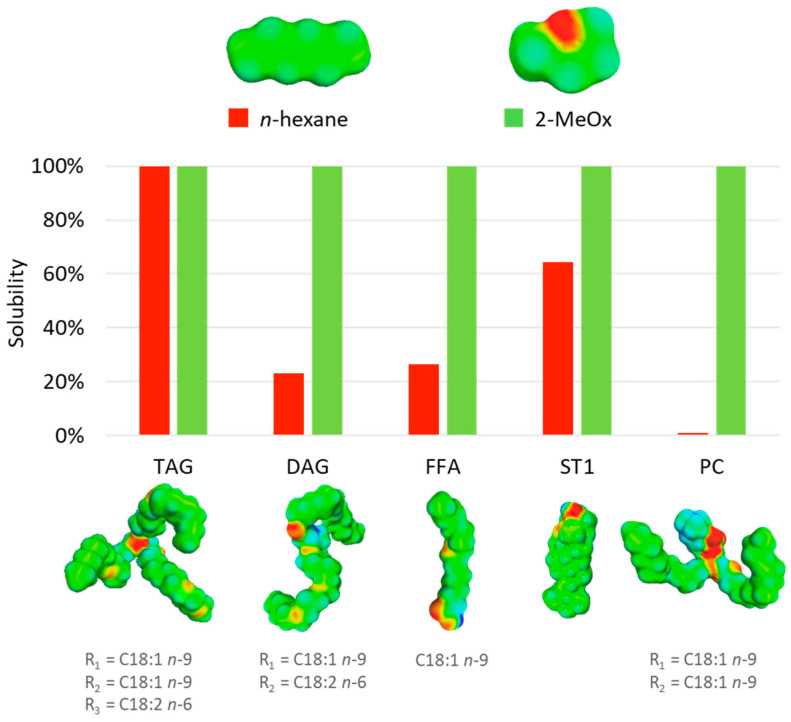
Representation of predicted solubilities of some lipids in *n*-hexane and 2-MeOx and their corresponding sigma surfaces, from [32,56]. TAG = Triglycerides, DAG = Diglycerides, FFA = Free fatty acid, ST1 = β-sitosterol, PC = Phosphatidylcholine. Rating: 0–20% bad solvent; 20–60% average solvent; 60–100% good solvent.

In order to assess the solubility of some neutral and polar lipids in 2-MeOx vs. hexane, COMO-RS calculations are shown in Figure 6. These results indicate that 2-MeOx could be as good as *n-*hexane in solubilizing lipids, and even better for polar lipids like phospholipids. The promising results obtained with predictive tools, were supported by several publications, as reported in Table 4.

A classical laboratory procedure to extract lipids with 2-MeOx is described by Cascant et al. [36]. First, the dried material is ground to a suitable size. Then, 25 to 30 g of raw material are weighed into a cellulose thimble and placed inside the extraction chamber of a Soxhlet apparatus. This latter is fitted with a condenser and put on a flask containing 300 mL of solvent. The sample is extracted under reflux for 8 h and after this time, the flask content is evaporated under reduced pressure.

Overall, the different works cited in Table 4 showed that considering oil recovery, 2-MeOx gave similar results compared to hexane in terms of lipid yield, fatty acid, and neutral lipid profile relative contents. Moreover, 2-MeOx is likely to provide a higher crude yield, due to the extraction of other compounds from the raw material. Indeed, higher proportions of phospholipids in oil were notably reported for different matrix [32,35,60]. It is worth mentioning that Saunois et al. patented the extraction of an oil or butter with high unsaponifiable content from a vegetable matrix or a micro-organism [67]. Beauduc et al. even patented the simultaneous extraction of lipophilic and polyphenolic compound from Douglas fir [61]. Bettaieb Rebey et al. found out that the oxidative stability of 2-MeOx extracted fennel and aniseed oils was significantly superior to hexane [58]. It is also interesting to note that some 2-MeOx extracted oils exhibited important anti-inflammatory activity [58,59]. Beside oil yield and composition, the protein quality of the residual meal or flour is also an important parameter to take care of when the matrix is defatted prior being used for food or feed. In this regard, Ravi et al. conducted a comparative study on the lipid extraction of black soldier fly larvae (BSFL) by *n*-hexane and 2-MeOx [56]. Protein quality of defatted BSFL flours was assessed through different parameters, including KOH solubility and protein dispersibility index (PDI). They found that the investigated protein quality parameters were slightly better in 2-MeOx defatted flour than the *n*-hexane defatted flour.

About the process itself, extractions using 2-MeOx were further examined with the help of kinetic studies by Sicaire et al. and Ravi et al. [31,56]. They observed the same trend over the extractions of rapeseed and BSFL oils: 2-MeOx is faster than hexane under the same conditions. Additional investigations would be of great help to understand this speed difference, but it could partly be due to a most effective cell disruption, as seen by Wan Mahmood et al. [64], or thanks to the partial miscibility with water that could help the solvent penetration into the solids.

With such promising results, the possibility of using 2-MeOx in replacement of hexane for the extraction of lipids was investigated with industrial simulations and even on a larger scale by Sicaire et al. [31], as discussed further in part 9. Indeed, hexane and 2-MeOx were tested on pressed rapeseed cake using a 6 L percolation extractor. The extraction was conducted by doing five washings of 30 min with fresh solvent. Residual oil content in the meal were 1.8% for hexane and 0.8% for 2-MeOx.

### 4.4. Miscellaneous

In addition to the above-mentioned compounds, Bundeesomchok et al. successfully extracted α-mangostin, a bioactive molecule, from *Garcinia mangostana* (mangosteen) with 2-MeOx [68].

## 5. Toxicity

2-MeOx has been used for more than a decade as a solvent for chemical synthesis and especially for the production of active pharmaceutical ingredients. It is now expanding into cosmetic and food extraction applications. The increasing use of 2-MeOx results in the potential exposure of workers and in the presence of limited but unavoidable residues in the finished products. Therefore, it is very important to establish a clear toxicological profile to warrant a safe use for workers, consumers, and the environment. In Europe, solvent producers and importers must comply with the REACH regulation (EC) No 1907/2006 by evaluating, among other things, the solvents toxicological profile, including acute, subchronic, and geno-toxicity. Data for 2-MeOx and hexane can be found on the ECHA’s website (European Chemicals Agency) [69]. A summary of the mandatory toxicological studies of 2-MeOx is shown hereafter.

### 5.1. Toxicological Data Collection

Henderson and Gurule reported the toxicokinetics of 2-MeOx in rats and mice in 2007 [70]. They concluded that the product had an oral absorption rate of 93%, rapid metabolism and excretion, and low bioaccumulation potential. Excretion was mostly via exhaled carbon dioxide and urine. Following oral exposure, the highest levels of radio activity were found in the kidney, but no tissue accumulated the radiolabel 2-MeOx in either species. The studies confirmed that, following oral exposure in the mouse or rat, there was almost complete absorption, making both species good models for the assessment of systemic toxicity.

### 5.2. Acute Toxicity

2-MeOx has of low acute toxicity via oral route (ingestion) and is essentially non-toxic following dermal or inhalation exposure (Table 5). It may cause severe eye damage and skin irritation but it is not a skin sensitizer.

The acute toxicity data is useful for the classification of the substance and the risk assessment of workers who are potentially exposed to the bulk material. 2-MeOx is classified for the following acute health hazards in accordance with Regulation (EC) No. 1272/2008:

- Category 3 Acute Toxicity, H301: Toxic if swallowed
- Category 1 Eye Damage, H318: Causes serious eye damage
- Category 2 Skin Irritant, H315: Causes skin irritation

### 5.3. The Subchronic Toxicity

The subacute toxicity of 2-MeOx has been investigated in rats via inhalation and ingestion (Table 6). There are two oral studies. The publication by Antonucci et al. [78] uses only a high dose level of 26 mg/kg/day and this is also identified as the no-observed-adverse-effect level (NOAEL); there is limited information available for this study. In 2017, the publication by Parris derives a NOAEL of 250 mg/kg/day; based on a good laboratory practice (GLP) study conducted to support the use of 2-MeOx as a solvent for pharmaceutical active ingredients manufacture [79]. There is no access to the full report but detailed group mean data is presented for the parameters assessed in a peer reviewed journal and results are highly reliable. In addition, this data from the oral study is most relevant to human risk assessment from dietary exposure.

In the oral study [79], the dose of 1000 mg/kg/day was associated with a slight decrease in male weight gain and, in both genders, effects associated with liver (increased liver weight, minimal/mild centrilobular hypertrophy, and increased serum cholesterol). At 500 mg/kg/day, there was a slight increase in liver weight, but no corresponding pathology. Otherwise these high dose levels were well tolerated and clinical signs were restricted to pre and post dose salivation in limited animals at both dose levels. It is not surprising to see effects in the liver at high doses; it has previously been shown that 93–100% of 2-MeOx is absorbed following oral administration [70] and the effects may be due to first pass metabolism and is considered an adaptive change; 250 mg/kg/day was a clear NOAEL for oral exposure.

Following nose-only inhalation exposure for 13 weeks, 10.0 mg/L was well tolerated and considered to be the no-observed-adverse-effect concentration (NOAEC). This dose level was associated with some effects that were considered to be none adverse and included transient unsteady gait and excessive salivation which were observed at routine observations; during the functional observation battery in week 13 abnormal gait/posture was also noted in females at this dose level. Liver weight was also slightly elevated at 4.5 and 10 mg/L, but there was no corresponding histopathology and the observation was again considered to be non-adverse. Although it was not quantified, inhalation exposure may transiently increase the systemic exposure to 2-MeOx, compared to oral exposure where most 2-MeOx must first pass through the liver where it is metabolised before general systemic exposure. Clinical signs following oral exposure to 1000 mg/kg/day were restricted to excessive salvation, and the observation of unsteady gait following inhalation exposure is without relevance when considering oral exposure in man. Taking into consideration all studies, the oral NOAEL is 250 mg/kg/day. Toxicokinetic assessment confirms that 2-MeOx is readily absorbed from the gastrointestinal tract and therefore effects of unabsorbed material do not need to be investigated.

### 5.4. Genotoxicity

The genotoxicity of 2-MeOx has been investigated by the National Toxicology Program (NTP) and by the pharmaceutical company Merck [81]. 2-MeOx was not genotoxic in bacterial or mammalian cells in vitro, nor in vivo tests (Table 7).

## 6. Solvent Regulations

### 6.1. Worker Safety

In the European Union (EU), the safety of workers and consumers for technical applications is regulated by REACH and 2-MeOx has been registered according to Regulation EC (No) 1907/2006. It is registered for annual quantities above 1000 tons for its use as a solvent for chemical synthesis including fine chemicals, agrochemicals, and pharmaceuticals. A comprehensive hazard identification has been conducted as part of this process.

The submitted hazard classification, made according to regulation EC (No) 1272/2008 on the classification, labelling and packaging of substances and mixtures (CLP Regulation) was:

- Flammable liquid category 2  H225: Highly flammable liquid and vapor.
- Acute Toxicity category 4   H302: Harmful if swallowed.
- Skin Irritant category 2    H315: Causes skin irritation.
- Eye Damage category 1    H318: Causes serious eye damage.

### 6.2. Pharmaceutical Applications

The maximum solvent residual in the pharmaceutical product is regulated worldwide by the document “Impurities: Guideline for residual solvents Q3C”. As long as the toxicological solvent database is not sufficient, the product remains in a “non-classified” list.

Based on the above mentioned key studies, the draft version of the 8th revision of the International Council for Harmonization (ICH) Q3C, issued in 2020, and currently under public consultation, proposes a permitted daily exposure for human (PDE) for 2-MeOx (2-MeTHF) of 50 mg/day. The document, which results from the discussion of an expert working group, also recommend that 2-MeOx would be placed into Class 3, “Solvents with low toxic potential”. This conclusion was based on the sub-chronic oral study reported by Parris in 2017 [79]. As hexane is generally the reference solvent for lipophilic extraction, it is interesting to compare this classification with the one of hexane in the Q3C Guideline for residual solvents (Table 8).

### 6.3. Cosmetic Applications

Cosmetic application often relies on toxicological assessment on published studies and pharmaceutical framework.

### 6.4. Food Applications

There is no worldwide regulation for solvent residue in food. Each country/territory has its own system, classification, and limits. As an example, Table 9 shows the regulatory body of different territories and limits in food for technical hexane.

2-MeOx is not registered for food application yet, the regulatory dossier is currently under review by the European Food Safety Authority (EFSA). Based on laboratory and scale-up tests, we anticipate that the residue in food will be equal to or less than the tolerated hexane residue in the European regulation.

## 7. Environmental Impacts of 2-Methyloxolane

Regarding environmental impacts, 2-MeOx offers a renewable alternative, reducing the need for non-renewable petroleum-based chemicals. Biobased products, through petroleum displacement, are known to play an increasingly important role in reducing greenhouse gas emissions that exacerbate global climate change [84].

**Figure 7:**
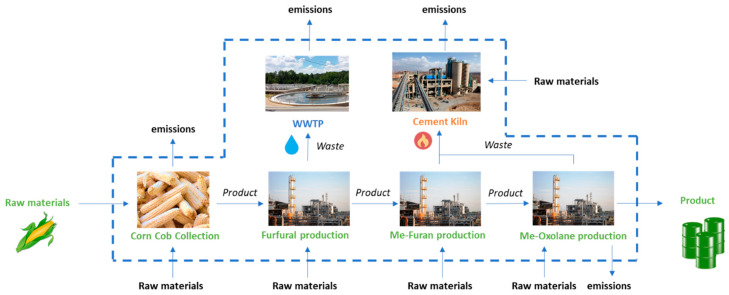
Life cycle assessment boundaries for 2-MeOx.

However, turning a molecule into a “green” carbon origin is not necessarily sufficient to reduce environmental impact, especially CO_2_ emission. Therefore, despite this high biobased content, a deeper analysis has been initiated back in 2012 to determine the global impact of 2-MeOx on carbon emissions. A Life Cycle Inventory (LCI) has been conducted by Slater et al. [85] to determine the cradle to gate life cycle emissions (Figure 7). Analyses have been performed using different methods to show greenness in both its production and industrial use.

**Figure 8:**
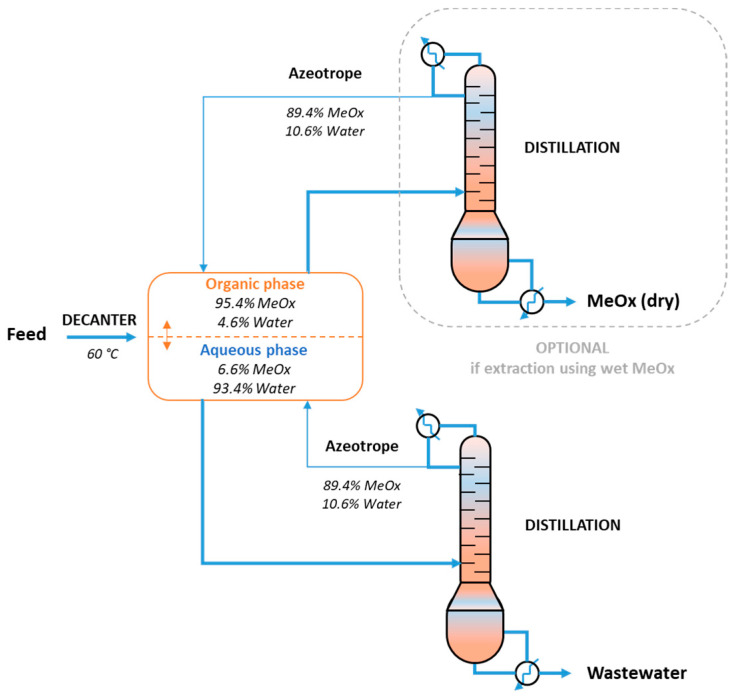
Dry 2-MeOx recovery process.

Using this approach, the overall life cycle emissions for production of 1 kg of 2-MeOx were determined to be 0.191 kg, including 0.150 kg of CO_2_ coming from the furfural production. Life cycle emissions generated to produce the starting block (furfural) represents most of the overall environmental impact (78%), the rest being generated during transformation into 2-MeOx. Compared to typically used solvents such as hexane, the evaluation showed that using 2-MeOx in an industrial scenario results in a 97% reduction in CO_2_ emissions. In case of solvent recycling (to dryness), the CO_2_ emissions of 2-MeOx increase to 0.247 kg of CO_2_ by kg of solvent (see distillation step, Figure 8), but it is greatly compensated by the reduction of the solvent consumption. However, it must be noted that for high-volume products in continuous processes (for instance, oils and fats), the 2-MeOx can be recycled without distillation (until dryness) by simple decantation of the condensed solvent-water azeotrope. In this case, the extraction is carried out with 95–96% purity 2-MeOx (+ water). Only the water phase recovered from the decantation has to pass through a distillation column before it is discharged, to recover the solvent dissolved in the aqueous phase (up to 14% at 20 °C).

**Figure 9:**
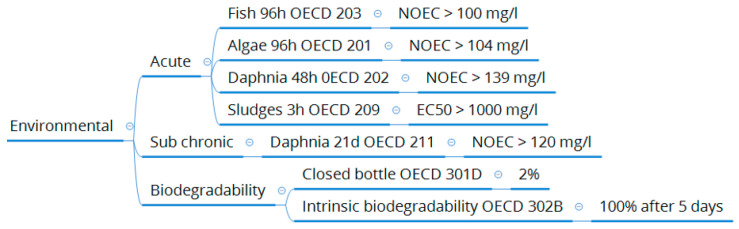
Eco-toxicological tests summary for 2-MeOx.

Most recently, in 2019, an eco-toxicological profile (Figure 9) has been carried out, as part of a REACH evaluation process, to determine environmental impact of 2-MeOx, if released in the environment. Those tests have been done according to international guidelines, under OECD references and revealed the innocuity of the solvent towards the environment and especially in water. In addition, its biodegradability has been proven after 5 days and compost testing according to watercress test showed no phytotoxicity for the product. As short-term EC50 (half maximal effective concentration) and long-term NOEC values are above 100 mg/L, 2-MeOx is not classified in the EU according to the regulation (EC) No. 1272/2008, while *n*-hexane is classified chronic category 3 (toxic to aquatic life with long lasting effects).

## 8. Economic Viability of 2-MeOx in Extraction Processes

Every industrial process needs to consider optimization of all steps in regards of the final market segment for the end-products. This becomes even more of importance in large scale continuous processes where every detail counts; 2-methyoxolane is so far to be considered as a specialty solvent in regards of its sales price range which is in the 7–9 €/kg range (Pennakem LLC, 2020), despite its production in several thousand tons a year—in comparison, food grade Hexane price is comprised in the 0.80–1.00 €/kg range and produced in millions of tons a year. Nevertheless, despite this significant price difference, 2-MeOx shows competitive edge when it comes to the economical equation of extraction processes; obviously if recycling is considered, and extraction process optimized with low solvent losses (i.e., lower than 1 kg per ton of extracted biomass/seeds). This has been demonstrated on few seeds currently extracted continuously using hexane. In accordance with the existing literature (see part 4), recent results we obtained at laboratory and pilot scale reported higher yields of extraction with lower residual oil in the meals which benefit for the whole mass balance evaluation and economical calculations. The difference in costs becomes negligible when it comes to evaluate all benefits of using an eco-friendly and safe solution to replace hexane in an optimized set-up.

Table 10 shows an example of preliminary calculations for canola (rapeseed) extraction. Some assumptions are coming from industry standards and especially for solvent loses in operation units that are set for the most modern ones, at 0.3 kg per ton of extracted seeds. Those losses are found in vapor and wastewater emissions, as well as residues in final products (oil and meal). Seed losses, estimated at 36 kg per ton, come from first crushing/pressing and preparation steps before solvent extraction and correspond to standards observed in the industry. Oil residual in the meal for hexane extraction could vary but standard industry average is found at 1.0–1.5%, whereas residual oil obtained with 2-MeOx usually vary between 0.5–1.0%. Sicaire et al. [31] confirmed part of those premises and run comparisons on various parameters including kinetics of extraction, playing an important role for productivity. As a result, and also thanks to the possibility to run at an higher extraction temperature (65 °C instead of 55 °C for hexane), we might have an improvement on extraction production cycle time impacting the overall process costs and converted into a gain of 0.9€ per ton of seeds. On the other hand, the simulation anticipates an additional steam consumption because of the enthalpy of vaporization difference between hexane (334 kJ/kg) and 2-MeOx (364 kJ/kg), as well as their boiling point difference, being 61 °C for hexane/water azeotrope and 69 °C for 2-MeOx/water azeotrope. The study reports the total energy consumption—heat supply or steam production injected—in the process and especially at the desolventization and recycling steps, requiring the major part of the energy consumed throughout the whole process for solvent vaporization, removal, and recovery. After various trials and optimizations, this impact has been calculated to be 1.1€ per ton of seeds (Table 10). Those key costs of the solvent extraction process have been included in the global cost evaluation as reported in the above chart showing finally an impact of only 0.47 € per ton of seeds extracted. This slight difference is minimal and could easily be compensated by applying a slight price premium on sales price of the edible oil. Less than one cent per kilo, representing 1% of premium, allows to even generate more benefits per ton of seeds, as reported in the last column of the chart.

Additional comparison could be done with Organic certified processes, such as mechanical pressing. In such cases, best case scenarios for oilseeds (double press) leave behind 8% oil in the meal, impacting the overall cost balance of the process more than 2-MeOx. Based on similar premises than in the above chart, earning difference compared to hexane reference will land at 18 € per ton of extracted seeds. This cost simulation must of course be updated as the scale-up will go along. Indeed, it is well known that when you carry out a change in a large highly optimized continuous process (and the oilseed hexane extraction process has been optimized since 1950), temporary additional production costs must be anticipated until the new optimum operating point is found. However, Table 10 calculations show that it should be possible to produce hexane free clean oil, plant protein, and feed meal with no major production cost change, once the process optimization will be over.

Beside the very large scale, highly optimized, and oilseed crushing industry, many other smaller scale extraction applications exist. There are numerous high added value compounds that are made in limited quantities and cannot justify a dedicated large-scale plant. Those species and compounds need to be made and produced either in multi-purposes and multi-solvent continuous facilities or in batch extractors. For such processes, 2-MeOx appears as well as an interesting option for the companies who want to get rid of petrochemicals residues in their product or claim a clean label production. As explained previously, hexane is widely used for flavor and fragrance production, recovered after the extraction and may be reused in further operations. In a very similar way, 2-MeOx could be recovered and reused allowing cost optimization on solvent losses, as well as on waste disposal. In any case, even if the solvent is not recycled, its disposal would be cheaper than the disposable of hazardous petro-based solvents, such as hexane.

## 9. Scale-Up

As exposed previously, many potential alternatives to hexane have been reviewed since the neurotoxicity of this solvent was discovered. Some solutions, such as supercritical CO_2_ or ethanol, offer today credible alternatives for small scale or high value-added extraction. However, none ever managed to compete with the large-scale continuous oilseeds crushing facilities. A typical oilseed crushing plant processes from 0.5 to 20 ktons of seeds/day. The oil and feed meals are commodities therefore the market is highly competitive and the margins are low. Even if the use of the 2-MeOx could open new higher value markets, such as organic oils for cosmetics, or clean label edible oils, the industrial players are asking for evidences that the good results obtained in the laboratory can be scaled up. To do so, we carried out several scale-up trials:

1. Multi-stages batch extractions (80 kg) on different seeds (soy, rapeseed, and sunflower). These tests allowed to refine the oil and confirm its quality for edible purposes. We also checked the quality of the proteins (typically, soy white flakes).
2. A continuous test on soybeans at Crown Iron Works facility (Minneapolis, MN, US) [86]. We produced about 150 kg of soy white flakes over 5 h on a percolation extractor (Crown Model III); 2-MeOx was compared with hexane using similar extraction conditions, both solvents achieved a residual oil content of 0.75%.
3. A continuous test on canola at ENAT International (Salamanca, Mexico).

### 9.1. Large-Scale Trial

**Figure 10:**
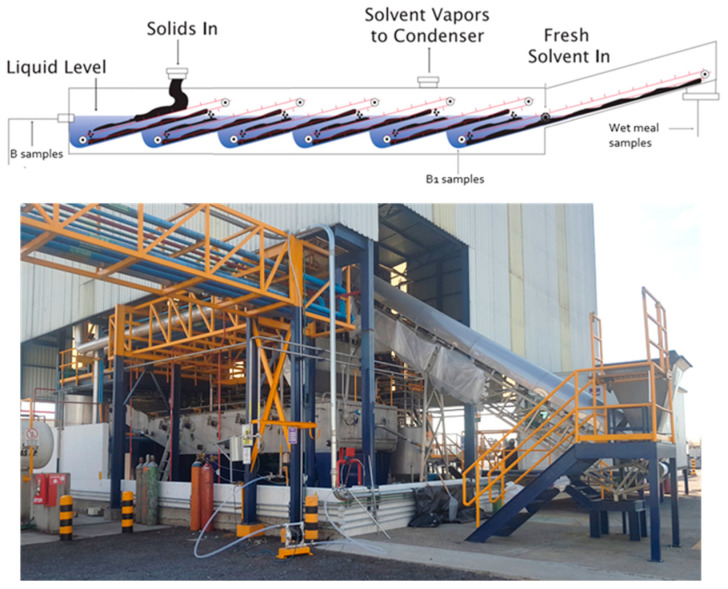
Counter-current extractor Crown MIV, immersion type (Crown Iron Works, Blaine, MN, USA) installed at Enat Internationalfacility (Salamanca, Mexico).

**Figure 11:**
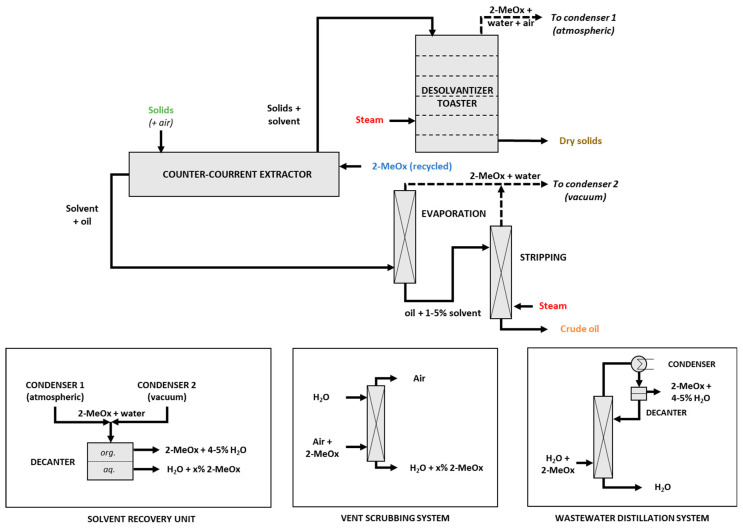
Simplified process diagram of the ENAT International facility (Salamenca, Mexico).

Recently, we successfully extracted more than 46 tons of canola press cake (22.8% oil) at 340 kg/h, including solvent recycling by simple decantation of the condensates [86]. This test was based on an immersion extractor (Figure 10), a desolventizer–toaster at atmospheric pressure of Schumacher type, a vacuum distillation installation consisting of a single-effect rising flow evaporator and a live steam stripper (Figure 11). All gas streams, equipment vents, and vapors generated during oil and seedcake desolventizing were condensed in two stages (not shown in Figure 10) and sent to the decanter. Condensates were decantated to obtain 2 phases: a first organic, mainly 2-MeOx (+4–5% water), reusable as is for extraction, and a second aqueous, sent in azeotropic distillation, to optimize the recovery of the solvent. An absorption column was used after the last step of condensation to reduce solvent loss and limit VOC emissions.

We produced a properly defatted (residual oil = 0.3%) and desolventized canola meal (residual solvent = 10 ppm). The optimized solvent/raw material feed ratio was 1.3/1 *w/w*, for a final oil concentration in the solvent of 20–25% oil. After a two steps desolventization, the solvent residue in the oil was between 0.5 and 1.5%; thus, a third step should allow to drop to the usual 50 ppm on crude oil. All gas streams, equipment vents, and vapors generated during oil and seedcake desolventizing were condensed in two stages and sent to the decanter. Condensates were decantated to obtain 2 phases: a first organic, mainly 2-MeOx (+4–5% water), reusable as is for extraction, and a second aqueous, sent in azeotropic distillation to optimize the recovery of the solvent. An absorption column was used after the last step of condensation to reduce solvent loss and limit VOC emissions. A simplified process diagram of an extraction facility converted for using 2-MeOx (ENAT International, Salamanca, Mexico) is shown Figure 11. Finally, in order to support the scale up process, we used the data we obtained during this trial to develop an oilseed plant simulation on a chemical process software CHEMCAD (version 7.1.8, Chemstations Inc., US). That simulation will help us to identify optimal working parameters and solve any bottleneck for further plants conversions.

### 9.2. Further Scale-Up

In order to support the scale up process for further plant conversions, we used a four-step systematical approach to secure the implementation of the 2-MeOx in an existing facility:

1. Preliminary diagnosis (on site visit and plant parameters collection). We issued a report where we list the needed adaptations.
2. Plant simulation. Estimation of the modification capital expenses and plant future operating costs.
3. Plant modification and run preparation
4. Run, data collection, and analysis of the results for further process optimization.

#### 9.2.1. Preliminary Diagnosis

This diagnosis consists in:

- Checking the overall plant condition and environment, safety culture, and risk prevention
- Checking the gasket compatibility: as 2-MeOx is a highly efficient solvent, some plastics or elastomer materials are not compatible.
- Collecting the plant process diagram and parameters necessary for the simulation.
- Identifying the potential sensors and sampling points for monitoring of the key parameters during the run.

#### 9.2.2. Plant Modification

The substitution of hexane by 2-MeOx does not require substantial modifications, unlike other solvents such as supercritical CO_2_ or liquefied gases, the only adaptations concern:

- Replacement of incompatible polymeric materials presents in seals, pump seals, conveyor belts, sight glasses, by per-fluorinated materials (PTFE, FFKM, etc.)
- Replacement of the wastewater boiler by an azeotropic distillation column to maximize the recovery of the solvent present in the water phase, before the water discharge or recycling.
- Setup a thermo-controlled decantation tank to reduce the quantity of 2-MeOx in the water phase (solubility in water drops from 14% at 20 °C to only 6.6% at 60 °C).
- Conversion of the vent solvent recovery system (Mineral Oil Absorption System). The petroleum-based light mineral oil can be replaced by cold water. Thus, the entire oilseed crushing facility would be free from petrochemical fluid and the risk of contamination of the edible oil by mineral oil would be removed.

## 10. Societal Impacts

**Figure 12:**
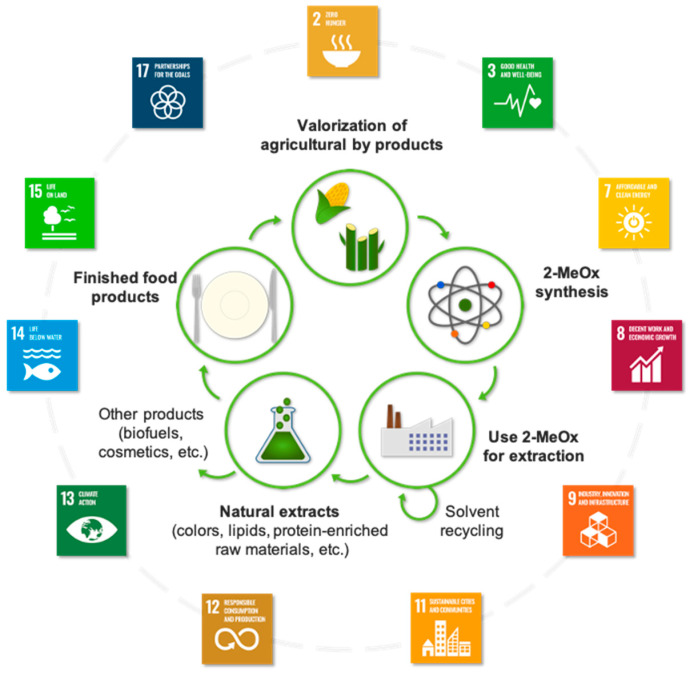
2-MeOx chain value and its potential positive contributions to sustainable development goals (SDGs).

The Agenda 2030 issued by the United Nations (UN) in 2015 emphasized the importance of sustainability, whether through its ecological, economic, or social dimensions. With the aim of addressing the daunting challenges that the world is currently facing, 17 Sustainable Development Goals (SDGs) were adopted. The SDGs represent global guidelines for both public and private actors that should enable overcoming poverty, hunger, and inequalities, mitigating environmental degradation and climate change and boosting economic growth and global development. As illustrated in Figure 12, 2-MeOx can contribute to meet 11 of the 17 SDGs all along its value chain.

First, 2-MeOx can align with SDG 2 “Zero Hunger”. The FAO/SOFI 2020 report related to “Food security and nutrition in the world” explains that 3 Billion people cannot afford a balance diet with particularly enough lipid, protein and fibers [87]. Unfortunately, today, the world food security in oil and protein depends on hexane, a petrochemical compound. Without hexane, the world oil production would drop of 20%, because the mechanical press cannot technically recover all the oil. Moreover, mechanical press produces a fat meal which has a low stability and get rancid after 3 months, generating food waste. There is currently no viable alternative to maximize the oil recovery, produce well defatted high stability protein meal, and make the most out of our valuable agricultural resources. In this context, 2-MeOx could offer an alternative, keeping all the hexane technical advantages with additional safety and sustainability. It could also allow to develop the use of the meal protein for human food instead of giving most of them to the feedstock, because of their high hexane content.

Then, all the natural extracts obtained with this bio-based solvent are free of neurotoxic and reprotoxic solvent residues and can therefore bring a positive contribution to SDG 3 “Good Health and Well-Being”. Some part of the non-edible agricultural by-products used to make 2-MeOx, as well as the lipid extracts, can be used as clean power sources which is in line with SDG 7 “Affordable and Clean Energy”. By replacing hazardous solvents by 2-MeOx, the working conditions of industrial workers could be greatly improved. This consideration meets the SDG 8 “Decent Work and Economic Growth”. As suggested by SDG 9 “Industry, Innovation and Infrastructure”, the development of 2-MeOx for extraction at industrial scale will require more solvent production capacity and open new market for hemicelluloses-rich by-products. 2-MeOx can meet SDG 11 “Sustainable Cities and Communities”, as the substitution of hexane by the 2-MeOx will reduce the risks of the oilseeds crushing plants for the local communities. Today, in Europe, because of the hexane toxicity for the environment, these facilities are within the Seveso regulation. They will exit that regulation with the 2-MeOx.

Because it is an easy-recyclable bio-based solvent, the use of 2-MeOx and derived products is in accordance with SDG 12 “Responsible Consumption and Production”. Replacing hazardous petrochemical solvents by 2-MeOx would drastically reduce the global dependence on fossil energy and CO_2_ emissions, limit health-harmful air and water emissions, and promote the valorization of agricultural waste. These latter points show that 2-MeOx can align with SDG 13 “Climate Action”, SDG 14 “Life Below Water”, and SDG 15 “Life on Land”. From its production to the consumption of related products, 2-MeOx would involve various stakeholders that could cooperate and help meeting the SDGs together. These corporate actions would embody SDG 17 “Partnerships for the Goals”. Overall, the use of 2-MeOx as an alternative to replace petrochemical solvents would participate in enhancing the global sustainability of our societies. This perspective is in line with the challenges that the world is facing, and the SDGs presented by the UN in 2015.

## 11. Future Trends

**Figure 13:**
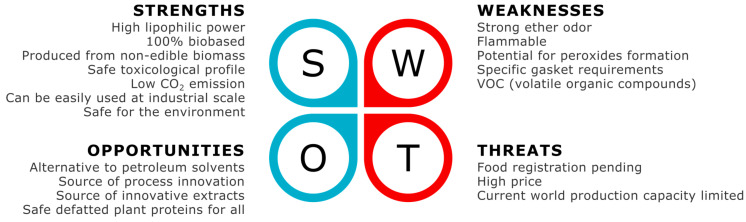
Strengths, weaknesses, opportunities, threats (SWOT) analysis (in 2020).

In this 21st century, extraction solvents have to be exclusively green, safe, and obtained only from renewable resources. Based on this literature review, it is clear that 2-MeOx is a credible alternative to hexane for the extraction of lipophilic natural products; 2-MeOx also opens new area of innovations, as it proved to be a viable and green alternative to hexane and related lipophilic petroleum solvents, but there are still some key challenges and barriers. Figure 13 is an attempt to summarize in a strengths, weaknesses, opportunities, threats (SWOT) analysis the current perspectives for 2-MeOx use as extraction solvent, from laboratory innovation to industrial applications. Briefly, even if 2-MeOx has some drawbacks (flammability, potential peroxides formation) they are totally compatible with large scale industrial use (2-MeOx is currently used in pharmaceuticals industry), and some of them are also a source of embedded safety (strong odor) for workers and consumers. Having interesting technical properties, a safe toxicological profile and low environmental impact, 2-MeOx has the potential to become the most credible alternative to hexane for extraction of natural products and can be a source of innovations (process and products). For example, 2-MeOx could be used in mixture with other bio-based solvents, such as ethanol or water, for specific extraction of more hydrophilic products (polar lipids, polyphenols, sugars, etc.).

At the time of writing, 2-MeOx can be only used for extraction of cosmetics (COSMOS label) or pharmaceuticals; applications for use as food/feed extraction solvent are now under review in several territories and the first approvals should be granted in 2021–2022. The possibility to use the 2-MeOx for the edible oil and protein extraction will bring a new credible industrial alternative to secure the world food supply, providing a clean and safe solution to produce oils and plant proteins and solving the current dependence to the petrochemical hexane.

## Figures and Tables

**Table 1:** Solvent properties of 2-MeOx vs hexane.

Properties/IUPAC Name	2-Methyloxolane [25,26]	Hexane (Extraction) [27]
Chemical structure	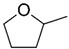	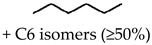
CAS number	96-47-9	64742-49-0 (extraction grade) 110-54-3 (*n*-hexane)
Synonyms	2-methyltetrahydrofuran; 2-MeOx; 2-MeTHF	-
Sourcing	Bio-based	Petro-sourced
Formula	C_5_H_10_O	C_6_H_14_
Molecular weight (g/mol)	86.1	86.2
Boiling point (°C)	80	66–70
Vapor pressure (kPa; 20 °C)	13.6	19.0
Vaporization enthalpy (kJ/kg)	364	334
Specific heat (kJ/kg.K; 25 °C)	1.8	2.2
Evaporation rate (BuAc) ^a^	4.2	8.0
Density (20 °C)	0.855	0.675
Viscosity (cP; 25 °C)	0.60	0.30
Electrical Conductivity (S/m; 25 °C)	8.10^−9^	1.10^−14^
Dielectric constant (25 °C)	7.0	1.9
Dipole moment (D)	1.38	0.09
Log *P*_o/w_	1.85	4.00
HSP ^b^ parameters (MPa ½)	δd = 16.8 δp = 4.8 δh = 4.6	δd = 15.0 δp = 0.0 δh = 0.0
Solubility in H_2_O (20 °C; %_w_)	14	1.10^−3^
H_2_O solubility (20 °C; %_w_)	4.4	9.10^−3^
Azeotropic point with H_2_O	71 °C/89%_w_	61.5 °C/94%_w_
Flash Point c.c. ^c^ (°C)	−11	−30
Auto-ignition temperature (°C)	270	225–375
Explosion range (vol%)	1.5–8.9%	1.1–7.4%

^a^ relatively to *n*-butylacetate; ^b^ Hansen Solubility Parameters; ^c^ c.c. = closed cup.

**Table 2:** Flavors and Fragrances extractions.

Matrix	Solvents ^a^	Extraction Conditions and Remarks ^b^	Ref.
*Ribes nigrum* L. (blackcurrant) buds	2-MeOx compared to *n-*hexane, α-pinene, BuOH, EtOH, EtOAc, EL, IPA, MeOAc	Reflux, BP, 2 h, 1:4 (*m/v*). 2-MeOx extract had a similar chemical composition to that of *n-*hexane one.	[25,29]
*Carum carvi* L. (caraway) seeds	2-MeOx compared to *n-*hexane, α-pinene, BuOH, DMC, EtOH, EtOAc, EL, IPA	Reflux, BP, 2 h, 1:10 (*m/v*). 2-MeOx gave the best limonene recovery among green solvents.	[30]
*Pistacia lentiscus* L. (lentisk) leaves	2-MeOx compared to hexane, CPME, DMC, EtOH, EtOAc, IPA	Reflux, BP, 2 h, 1:10 (*m/v*). 2-MeOx extraction yield was the third higher among green solvents.	[41]
*Citrus sinensis* L. (orange) peel waste	2-MeOx compared to hexane, CPME, DMC, EtOAc, EL, IPA, IPAc, MEK, PEG 300	Maceration in a shaking incubator, **70 °C**, **1.5 h**, **1:10** (*m/v*). 2-MeOx increased limonene yield by 40% compared to hexane.	[42]
*Humulus lupulus* L. (hop) cones	2-MeOx compared to hexane	Reflux, BP, 2 h, 1/10 (*m/v*). Soxhlet, 40–60 °C, 6 h, 1:6 (*m/v*). 2-MeOx and hexane extracts had similar olfactory profiles.	[40]

^a^ BuOH = Butanol, CPME = Cyclopentyl methyl ether, DMC = Dimethylcarbonate, EtOH = Ethanol, EtOAc = Ethyl acetate, EL = Ethyl lactate, IPA = Isopropyl alcohol, IPAc = Isopropryl acetate, MeOAc = Methyl acetate, MEK = Methyl ethyl ketone, PEG 300 = Polyethylene glycol 300. ^b^ Optimized conditions (when available) are in **bold**, BP = Boiling Point, given ratios are solid-to-solvent ratios.

**Table 3:** Color extractions.

Matrix	Solvents ^a^	Extraction Conditions and Remarks ^b^	Ref.
Plants			
*Daucus carota* L. (carrot) taproot	2-MeOx compared to *n-*hexane	Reflux, BP, 6 h. This kinetic study showed that extraction was faster with 2-MeOx than with *n-*hexane.	[25]
*Daucus carota* L. (carrot) taproot	2-MeOx compared to *n-*hexane, CPME, DMC, EtOAc, IPA	Reflux, BP, 1 h, 1:4 (*m/v*). Carotenoid yields (m% DM) were 65.8 and 55.8 for 2-MeOx and *n-*hexane, respectively corresponding to 80 and 68% recovery.	[46]
Microalgae			
*Chlorella vulgaris* Beij. dry and wet	2-MeOx compared to 2-MeOx/EtOH (1:1)	ASE ^c^, 103 bars, 110 °C, 30 min. Pure 2-MeOx enabled 38 and 45% carotenoid recovery from dry and wet biomass.	[47]
*Haematococcus pluvialis* Flot. culture	2-MeOx compared to almond oil, BuOH, cyclohexane, DEC, DMC, EtOAc, IAA, MIBK	Liquid/liquid extraction, RT, 30 min, 3:1 (*v/v*). 2-MeOx performance was the second highest with more than 80% astaxanthin recovery.	[48]

^a^ BuOH = Butanol, CPME = Cyclopentyl methyl ether, DEC = Diethylcarbonate, DMC = Dimethylcarbonate, EtOH = Ethanol, EtOAc = Ethyl acetate, IAA = Isoamyl alcohol, IPA = Isopropyl alcohol, MIBK = Methyl isobutyl ketone. ^b^ BP = Boiling Point, RT = Room Temperature, given ratios are either solid-to-solvent ratios or liquid-to-solvent ratios. ^c^ ASE = Accelerated Solvent Extraction.

**Table 4:** Lipid extractions.

Matrix	Solvents ^a^	Extraction Conditions and Remarks ^b^	Ref.
Plants			
*Brassica napus* L. (rape) seeds	2-MeOx compared to *n-*hexane, CPME, DMC, EtOH, EtOAc, IPA, limonene, p-cymene	Soxhlet, 40–60 °C, 8 h, 1:5 (*m/v*). 2-MeOx gave the highest lipid yield, 47.2% DM versus 46.7% for *n-*hexane.	[35]
*Brassica napus* L. (rape) seed cakes	2-MeOx compared to *n-*hexane	Soxhlet, 55 °C, 2 h, 1:5 (*m/v*). Maceration, 55 °C, 2 h, 1:5 (*m/v*). Pilot scale percolation, 55 °C, 5 × 30 min, 2:3 (*m/m*). The kinetic study showed that extraction was faster with 2-MeOx than with *n-*hexane.	[31]
*Phoenix dactylifera* L. (date palm) seeds	2-MeOx compared to *n-*hexane	Soxhlet, 40–60 °C, 8 h, 1:13 (*m/v*). Maceration, 40 °C, 30 min, 1:10 (*m/v*). Ultrasound, 20 kHz, 130 W, 40 °C, 30 min, 1:10 (*m/v*). Microwave, 20 MHz, 450 W, 40 °C, 30 min, 1:10 (*m/v*). The highest lipid yields were obtained by ultrasound method, with 5.57 and 6.18% for 2-MeOx and *n*-hexane.	[57]
*Pimpinella anisum* L. (anise) and *Foeniculum vulgare* Mill. (fennel) seeds	2-MeOx compared to *n-*hexane	Soxhlet, 40–60 °C, 8 h, 1:4 (*m/v*). 2-MeOx extracts showed the highest total phenolic content, strongest antioxidant activity and oxidative stability.	[58]
*Carum carvi* L. (caraway) seeds	2-MeOx compared to *n-*hexane	Soxhlet, 40–60 °C, 6 h, 1:4 (*m/v*). 2-MeOx extract had a higher total phenolic content and 2 times strongest antioxidant activity than *n*-hexane extract.	[59]
*Pistacia lentiscus* L. (lentisk) fruits	2-MeOx compared to hexane, CPME, DMC, EtOH, EtOAc, EL, IPA, limonene, α-pinene, p-cymene	Soxhlet, 40–60 °C, 8 h, 1:4 (*m/v*). 2-MeOx was found to be the best alternative solvent compared to hexane both qualitatively and quantitatively.	[60]
*Pseudotsuga menziesii* (Mirb.) Franco (Douglas fir)	2-MeOx as mono-solvent or in mixtures with EtOAc, EtOH and water	ASE ^c^, 100 bars, 50–90 °C, 3–45 min. In this invention 2-MeOx is used to extract simultaneously lipophilic and polyphenolic molecules.	[61]
*Opuntia ficus-indica* L. (prickly pear) seeds	2-MeOx compared to *n*-hexane	Soxhlet, 40–60 °C, 8 h, 1:10 (*m/v*). 2-MeOx gave the highest oil yield i.e., 9.55% versus 8.86% for *n-*hexane.	[62]
Oleaginous microalgae and microorganisms			
*Nannochloropsis* sp. D.J.Hibberd culture	2-MeOx compared to CHCl_3_, heptane, cyclohexane, toluene, CPME, DMC, EtOAc, MIBK, MTBE, limonene	Liquid/liquid extraction, 20 °C, 10 min, 2:1 (*v/v*). 2-MeOx extracted the most total fatty acids among green solvents.	[63]
*Chlorella vulgaris* Beij. and *Nannochloropsis* sp. D.J.Hibber	2-MeOx compared to hexane, CPME, EtOAc, EL	Soxhlet, 40–60 °C, 8 h, 1:100 (*m/v*). 2-MeOx provided twice extraction yield in comparison with hexane and showed the most effective cell disruption of biomass.	[64]
*Chlorella pyrenoidosa* Beij.	2-MeOx compared to pure *n-*hexane, CHCl_3_, IAA, and different mixtures of these solvents with water, EtOH and MeOH	Maceration, RT, 30 min, 1:10 (*m/v*). Bligh and Dyer, RT. Regarding maceration, 2-MeOx recovered 10.3% (lipids) vs 6.12% with *n*-hexane.	[65]
*Chlorella pyrenoidosa* Beij.	2-MeOx compared to pure hexane, CHCl_3_, CPME and different mixtures of these solvents with water, MeOH, IAA, and IPA	Soxhlet, 40–60 °C, 8 h, 1:4 (*m/v*). Single solvent maceration, RT, 45 min, 1:4 (*m/v*). Bligh and Dyer, Folch and Hara and Radin, RT. After maceration, 2-MeOx recovered 2.98% (lipids) while 0.96% with hexane.	[66]
*Yarrowia lipolytica* Van der Walt and Arx	2-MeOx compared to hexane CPME, DMC, EtOH, EtOAc, EL, IPA, limonene, α-pinene, p-cymene	Maceration, RT, 1h, 1:45 (*m/v*). 2-MeOx was identified among the most promising green solvent to replace hexane for extraction of microbial oils.	[32]
Animal sources			
*Salmo salar* L. (salmon fish)	2-MeOx compared to hexane CMPE, DMC, EtOH, EtOAc, IPA, limonene, p-cymene	Soxhlet, 40–60 °C, 8 h, 1:5 (*m/v*). 2-MeOx gave qualitatively and quantitatively (32.5%) similar oil than that of hexane.	[36]
*Hermetia illucens* L. (black soldier fly) larvae	2-MeOx compared to *n-*hexane	Soxhlet, 40–60 °C, 6 h, 1:10 (*m/v*). Maceration, 55 °C, 3 h, 1:10 (*m/v*). Multistage cross current industrial simulation, 55 °C, 3 × 60 min, 1:10 (*m/v*). 2-MeOx yielded more oil with enhanced bioactivity and the protein quality parameters of defatted flour were slightly better than with *n*-hexane.	[56]

^a^ CPME = Cyclopentyl methyl ether, DMC = Dimethylcarbonate, EtOH = Ethanol, EtOAc = Ethyl acetate, EL = Ethyl lactate, IAA = Isoamyl alcohol, IPA = Isopropyl alcohol, MeOH = Methanol, MIBK = Methyl isobutyl ketone, MTBE = Methyl tertbutyl ether. ^b^ RT = Room Temperature. ^c^ ASE = Accelerated Solvent Extraction.

**Table 5:** Summary of the studies assessing acute toxicity and irritation.

Endpoint	Method	Results	Ref.
Oral acute toxicity (rat)	Acute Oral Toxicity-Fixed Dose Method *OECD Guideline 420*	LD_50_: 300–2000 mg/kg bw (female)	[71]
Oral acute toxicity (rat)	*not current guideline*	LD_50_: 3800 mg/kg bw	[72]
Acute inhalation toxicity (rat)	*not current guideline*	LC_50_: 22 mg/L air	[72]
Acute dermal toxicity (rat)	Acute Dermal Toxicity *OECD Guideline 402*	LD_50_: >2000 mg/kg bw (male/female)	[73]
Acute dermal toxicity (rabbit)	*not current guideline*	LD_50_: 4500 mg/kg bw	[72]
Skin corrosion in vitro study	In Vitro Skin Corrosion: Human Skin Model Test *OECD Guideline 431*	Non-corrosive	[74]
Skin irritation in vitro study	In Vitro Skin Irritation *OECD Guideline 439*	Irritating Relative mean viability = 40.4%	[75]
Eye irritation ex-vivo study	Bovine Corneal Opacity and Permeability Test Method for Identifying Ocular Corrosives and Severe Irritants *OECD Guideline 437*	Corrosive/severe irritant	[76]
in vivo study Skin sensitization	Skin Sensitization: Local Lymph Node Assay *OECD Guideline 429*	Not sensitizing	[77]

OECD: Organization for Economic Co-operation and Development; LD50: Median lethal dose; bw = body weight.

**Table 6:** Summary of the subchronic toxicity, oral and inhalation.

End Point	Method	Results	Ref.
Subchronic toxicity, oral (rat)	subchronic ora l0, 80, 250, 500 and 1000 mg/kg/day Exposure: 3 months + 1-month recovery Equivalent to *OECD 408*	NOAEL: 250 mg/kg bw/day (male/female) increased liver weight and hypertrophy	[79]
	subchronic oral 26 mg/kg bw/day + unspecified lower doses Exposure: “approximately 3 months” (daily) Equivalent to *OECD 408*	NOAEL: 26 mg/kg bw/day (male/female) No toxicity observed	[78]
Subchronic toxicity, inhalation (rat)	subchronic inhalation 0, 2, 4.5 and 10 mg/L Exposure: 3 months (6 h/day for 5 days/week) Additional investigations (FOB, estrous cycle monitoring thyroid analysis, sperm analysis). *OECD 413*	NOAEC: 10 mg/L (male/female) some non-adverse transient clinical signs, and minor bodyweight and food consumption effects at 10 mg/L	[80]

NOAEL: No-observed-adverse-effect level; NOAEC: No-observed-adverse-effect concentration.

**Table 7:** Summary of genotoxicity studies.

End Point	Method	Results	Ref.
in vitro bacterial mutation	Bacterial reverse mutation assay (e.g., Ames test) (gene mutation) *S. typhimurium* Equivalent to *OECD Guideline 471*	Negative Test concentrations: 10–10,000 μg/plate	[81]
	Bacterial reverse mutation assay (e.g., Ames test) (gene mutation) *S. typhimurium* and *E. coli* *OECD Guideline 471*	Negative Test concentrations: up to 5490 µg/plate	[78]
in vitro mammalian mutation	Mammalian cell gene mutation assay (gene mutation) mouse lymphoma L5178Y cells Equivalent to *OECD Guideline 476*	Negative Test concentrations: 63.75–1020 μg/mL	[82]
	Mammalian cell gene mutation assay (gene mutation)-mouse lymphoma cells Equivalent to *OECD Guideline 476*	Negative Test concentrations: 1500–5000 μg/ml	[81]
in vitro mammalian micronucleus	Human lymphocytes (chromosome aberration) *OECD Guideline 487*	Negative Test concentrations: up to 10 mM	[83]
in vitro mammalian cytogenicity	*In vitro* mammalian chromosome aberration test (chromosome aberration) lymphocytes: peripheral human *OECD Guideline 473*	Negative Test concentrations: up to 10.7 mM	[78]
in-vivo micronucleus	Micronucleus assay (chromosome aberration)-rat male/female *OECD Guideline 474*	Negative Oral doses: up to 26 mg/kg/day	[78]

**Table 8:** Hexane versus 2-MeOx in the International Council for Harmonization (ICH) Q3(R8) draft.

	PDE (mg/day)	Maximum Residual Solvent	Classification
Hexane	2.9	290 ppm	Class 2: solvent to be limited
2-MeOx	50	According to GMP (up to 5000 ppm)	Class 3: low toxicity solvent

GMP: Good Manufacturing Practices.

**Table 9:** Different territories, different regulations.

Regulatory Body	Territory	Hexane for Food
FDA	USA	Hexane: no limit in oil and proteins Limits in flavors
FSANZ	Australia/New Zealand	Hexane: <20 ppm in all foods
JETRO	Japan	Hexane: <5 ppm in oils
Health Canada	Canada	Hexane: <10 ppm in oils and proteins
EFSA	Europe	Hexane: <1 ppm in oil and flavors and <10 ppm in food formulated with proteins

FDA: Food and Drugs Administration; FSANZ: Food Standards Australia New Zealand; JETRO: Japan External Trade Organization; EFSA: European Food Safety Authority.

**Table 10:** Turnover estimations for rapeseed oil extraction with standard residual oil observed in the meal.

Scenarios *	Hexane (Reference)	2-MeOx	2-MeOx (Oil with Premium Price)
Oil price (€/t)	710	710	717 (1% premium)
Seeds (kg)	1000	1000	1000
Preparation losses (kg)	36	36	36
% oil in meal	1.5%	0.8%	0.8%
Solvent consumption (kg)	0.30	0.30	0.30
Oil quantity (kg)	416	420	420
Oil recovery vs hexane		0.93%	0.93%
Meal quantity (kg)	548	544	544
Oil turnover (€)	295	298	301
Meal turnover (€)	126	125	125
Solvent cost (€)	0.0	−2.4	−2.4
Steam extra-cost (€)	0	−1.1	−1.1
Productivity gain (€)	0	0.9	0.9
Total turnover €/ton seeds	421.40	420.66	423.64
Delta vs hexane €/ton seeds	/	−0.47	+2.51

* The following assumptions were used: standard rapeseed oil price at 710 €/ton, standard rapeseed meal price at 230 €/ton, hexane price at 900 €/ton, 2-methyoxolane price at 8000 €/ton.
